# Foodscape: A scoping review and a research agenda for food security-related studies

**DOI:** 10.1371/journal.pone.0233218

**Published:** 2020-05-20

**Authors:** Simon Vonthron, Coline Perrin, Christophe-Toussaint Soulard

**Affiliations:** INNOVATION, Univ Montpellier, INRAE, CIRAD, Montpellier SupAgro, Montpellier, France; Uppsala University, SWEDEN

## Abstract

Since 1995, the term ‘foodscape’, a contraction of food and landscape, has been used in various research addressing social and spatial disparities in public health and food systems. This article presents a scoping review of the literature examining how this term is employed and framed. We searched publications using the term foodscape in the Web of Science Core Collection, MEDLINE, and Scopus databases. Analyzing 140 publications, we highlight four approaches to the foodscape: (i) Spatial approaches use statistics and spatial analysis to characterize the diversity of urban foodscapes and their impacts on diet and health, at city or neighborhood scales. (ii) Social and cultural approaches at the same scales show that foodscapes are socially shaped and highlight structural inequalities by combining qualitative case studies and quantitative surveys of food procurement practices. (iii) Behavioral approaches generally focus on indoor micro-scales, showing how consumer perceptions of foodscapes explain and determine food behaviors and food education. (iv) Systemic approaches contest the global corporate food regime and promote local, ethical, and sustainable food networks. Thus, although spatial analysis was the first approach to foodscapes, sociocultural, behavioral and systemic approaches are becoming more common. In the spatial approach, the term ‘foodscape’ is synonymous with ‘food environment’. In the three other approaches, ‘foodscape’ and ‘food environment’ are not synonymous. Scholars consider that the foodscape is not an environment external to individuals but a landscape including, perceived, and socially shaped by individuals and policies. They share a systemic way of thinking, considering culture and experience of food as key to improving our understanding of how food systems affect people. Foodscape studies principally address three issues: public health, social justice, and sustainability. The review concludes with a research agenda, arguing that people-based and place-based approaches need to be combined to tackle the complexity of the food-people-territory nexus.

## Introduction

As reported by the High Level Panel of Experts on Food Security and Nutrition (HLPE): “Every human being has the right to adequate food. However, the progressive realization of this right will not be achieved without more sustainable food systems that facilitate healthy and sustainable food choices” [1]. Like other authors [2,3], this report suggests that health and well-being are affected by the living environment (e.g. built, social, and food). Concerning food, the HLPE report defines the ‘food environment’ as “the physical, economic, political and socio-cultural context in which consumers engage with the food system to make their decisions about acquiring, preparing, and consuming food” [1]. Much of the scientific literature, including several recent reviews [4,5], defines the food environment with reference to the seminal conceptual framework of Glanz *et al*. [6]. These studies distinguish the community environment (types of outlets, accessibility), the consumer environment (within-store availability of healthy options, price, nutrition information), and the organizational food environment (home, school or work). The main research question addressed is the influence of food environment on diet and health [4,7]. Living in ‘food deserts’, i.e. in areas where physical access to grocery stores and supermarkets is limited [8,9], could hence be a health issue. Within the food environment field, scholars scrutinize environmental variables and try to distinguish their impact on food behaviors from individual variables (such as sociodemographic characteristics, psychosocial factors). They hence conceive the food environment primarily as a physical/external context in which individuals evolve.

Other scholars use the foodscape concept to address what appear to be similar food issues. This concept is etymologically defined by Adema [10] as “a marriage between food and landscape, both the conceptual notion (idea) of landscape and actual, physical landscapes”. The term ‘foodscape’ first appeared in academic literature in 1995 [11], and is increasingly encountered in the English-speaking literature. At first glance, this term may appear synonymous with ‘food environment’. However, no currently available systematic review of the literature has specifically looked at how ‘foodscape’ has been employed and framed.

The objective of our article is to clarify the definitions, uses, and utility of the term ‘foodscape’. Which scholars and approaches use it? Why do they use ‘foodscape’ rather than/ or together with the term ‘food environment’? And how does this foodscape concept contribute to current debates on food systems’ effects on people? We address these questions via a scoping review of the literature using the term ‘foodscape’. We first present our search strategy and criteria for selecting and analyzing 140 publications from international databases. Then, the results section distinguishes four approaches to the foodscape stemming from different research communities. Each approach includes subgroups addressing specific research questions. These results enable us to examine the two concepts of foodscapes and food environment, and to identify the unique features and added-value of the foodscape concept. We conclude with a future research agenda, arguing that the foodscape concept can help tackle the complexity of the food-people-territory nexus.

## Methods

### Search strategy

Our review follows the guidelines on Preferred Reporting Items for Systematic Reviews and Meta-Analyses extension for Scoping Reviews (PRISMA-ScR) [12]. The PRISMA-ScR checklist is available in S1 Checklist.

In July 2019, we searched three electronic databases: MEDLINE, Scopus and Web of Science Core Collection. In these databases, we looked for article titles, abstracts and keywords using the following query strings: “TITLE-ABS-KEY(foodscape*)” in Scopus and “TS = foodscape*” in MEDLINE and Web of Science Core Collection. We did not restrict to specific dates of publication or research areas.

After removal of duplicate records, only published peer-reviewed articles, proceedings papers in full text, books, book chapters and editorials were included. The following were excluded: (i) publications in which the term ‘foodscape’ refers to non-human food, (ii) publications in which the term ‘foodscape’ is used incidentally but is not a central theme, (iii) two publications, one in Slovenian, and another that could not be found on the website of the journal.

### Data collection and extraction

For each publication, a descriptive form was completed with the following items: title, type of publication, journal’s name (or book’s name for book chapters), authors, academic field of first author, year of publication, case study (‘yes’ or ‘no’), countries studied, methods, scale, research question, definition of ‘foodscape’ and/or authors referred to when defining it.

We extracted the academic fields of first authors and the countries of authors’ affiliation from the websites of their universities or institutes of affiliation. In cases where authors had several affiliations, we only considered the first indicated in the publication. We extracted all other data from readings of the publications themselves.

### Categorization of publications

This descriptive form was used to distinguish between approaches to ‘foodscape’ and to categorize publications accordingly. After a first screening of all the publications, we distinguished between approaches by grouping publications that: i) used a similar definition of ‘foodscape’ and referred to the same authors when defining it; and ii) shared similar methods. We then verified that such approaches actually reflected practice in existing research communities by looking at authors, journals, and academic fields. We therefore categorized all publications included in the scoping review according to these approaches. After a second reading of the publications classified under each approach, we listed the main research questions, which enabled us to distinguish subgroups of publications sharing similar objectives. Grouping criteria were refined through discussion and consensus among the authors. Then, the whole corpus of publications was categorized into approaches and subgroups by the first author. When a publication fell into several subgroups, its category was chosen by consensus among the authors.

## Results

### Corpus

**Fig 1:**
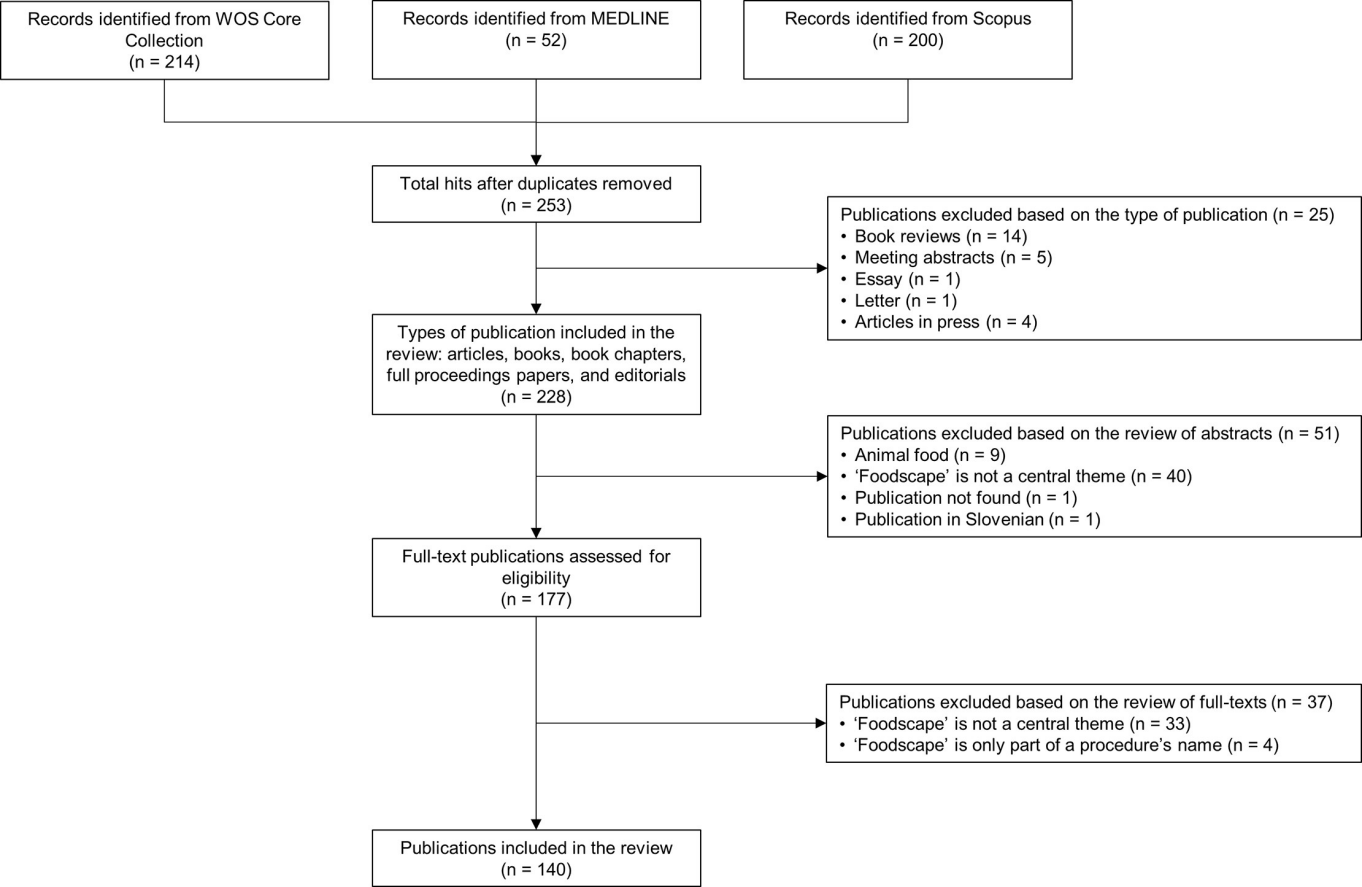
Flow diagram of the search process.

We identified 466 publications from the electronic databases since 1995 (date of the first occurrence of the term). A total of 326 publications were removed, 213 of them as duplicates identified through matching title, authors, year of publication and journal. Based on our eligibility criteria, a total of 140 publications are included in this review (Fig 1). The main characteristics of each included publication are available in S2.

## Publications reviewed: Type and geographical location

**Fig 2:**
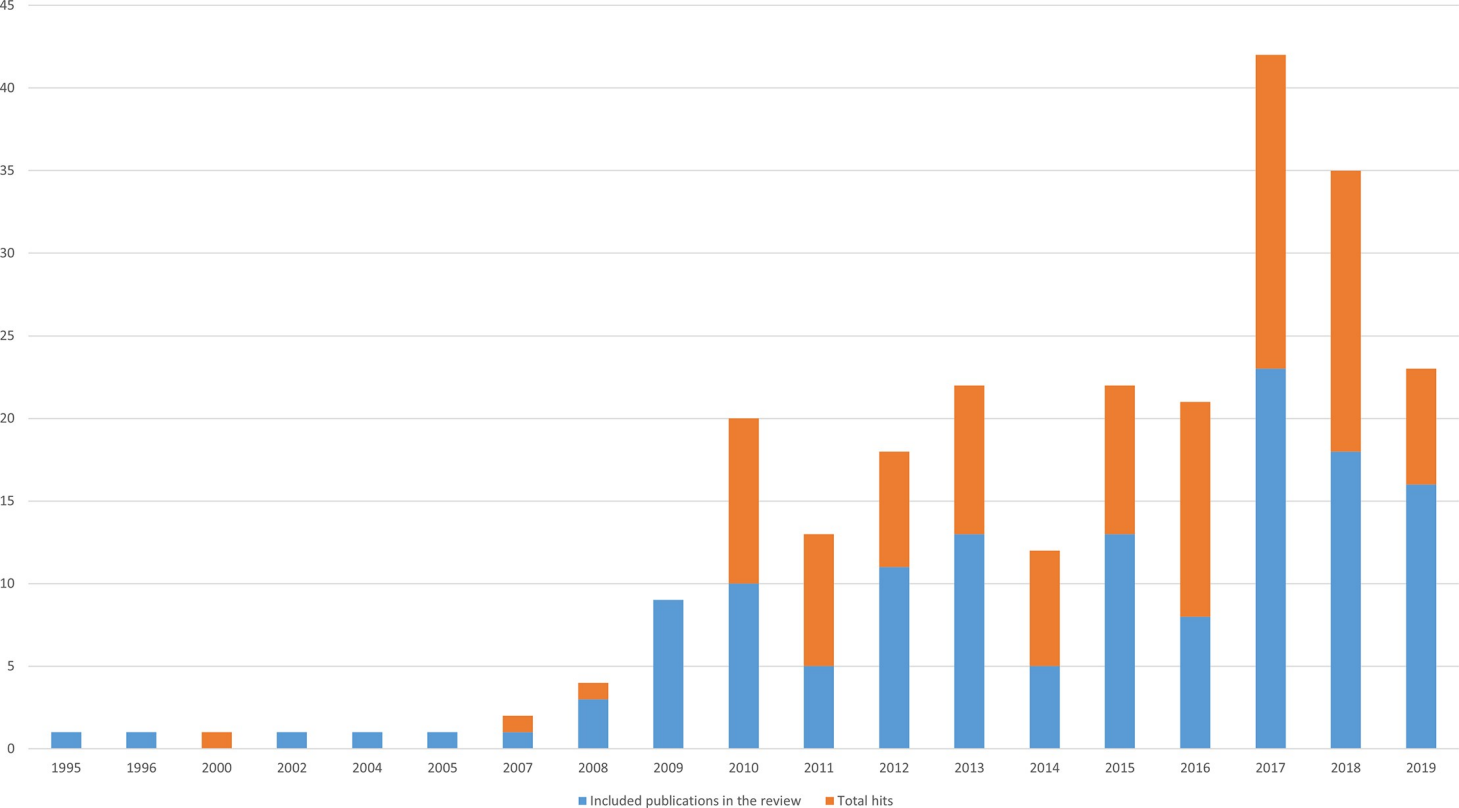
Number of publications using the term foodscape in the corpus of publications included in the review. 2019 includes from January to June.

**Fig 3:**
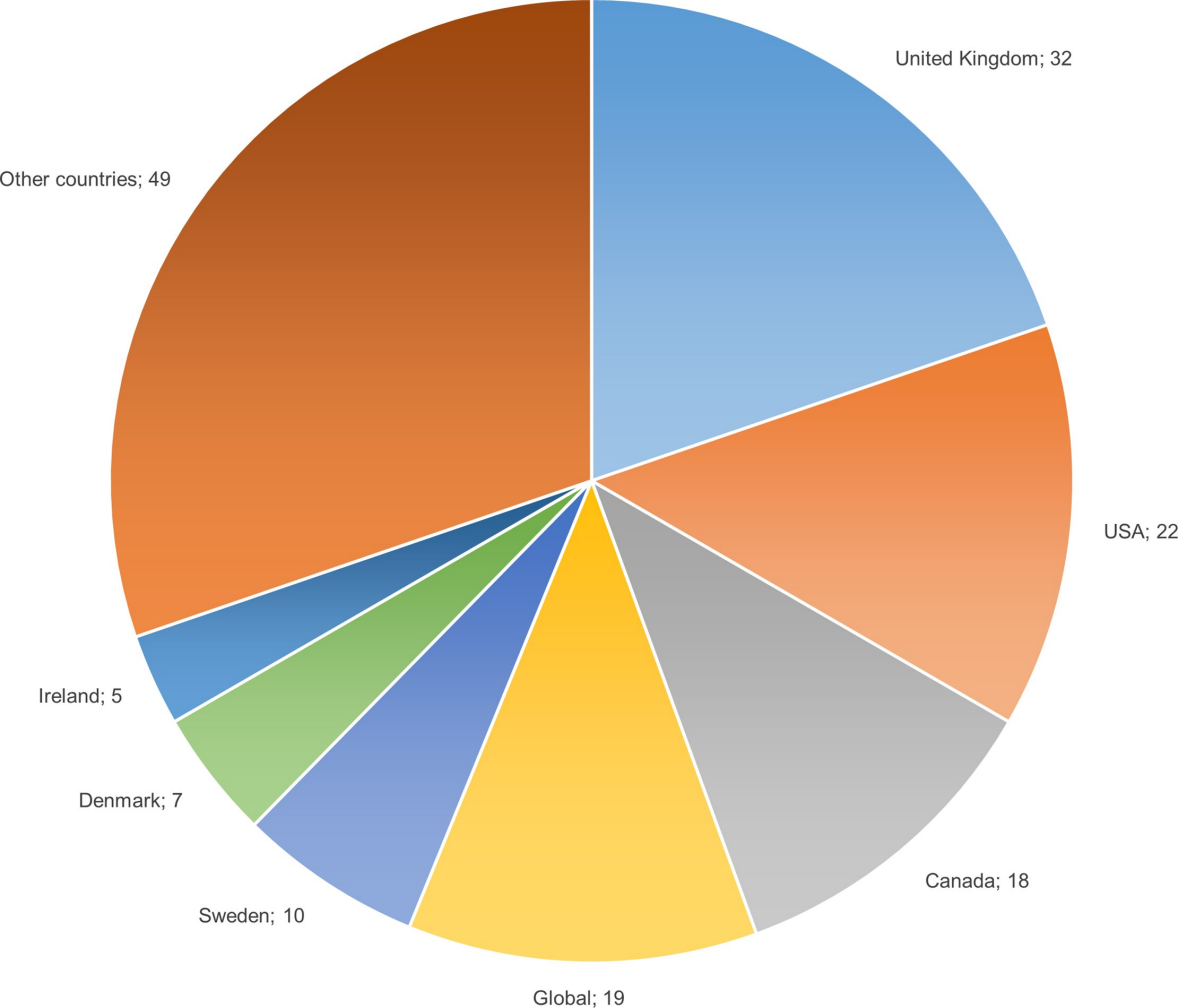
Countries studied in the publications of the corpus. Numbers refer to the number of studies on a country. Eleven publications include studies on different countries. ‘Other countries’ cover 35 countries and areas studied between 1 and 4 times.

The number of publications using the term ‘foodscape’ has grown since 2007 (Fig 2). Of the 140 publications included in this review, 116 are peer-reviewed articles. The corpus includes 344 authors, six of whom wrote more than three of the publications: Burgoine T. (7 articles), Lake A. (5), Kestens Y. (5), Sonnino R. (5), Cummins S. (4), and Morgan K. (4). 116 of the publications include case studies, mainly covering three geographical areas: USA and Canada (50 publications), United Kingdom and Ireland (37 publications) and Sweden and Denmark (17 publications) (Fig 3).

We distinguished four approaches to the foodscape: spatial, social and cultural, behavioral, and systemic (Table 1).

**Table 1:** Approaches and characteristics of included publications.

Approaches	Definitions of foodscape	Methods	Main research questions	Subgroups	Main academic fields	Main areas and countries	References
Spatial approaches	Spatial distribution of food outlets. Community nutrition environment	Statistics, (participatory) GIS, cross-sectional surveys, observations, store audits	How are food outlets spatially distributed? What are their dynamics?	Characterizing the diversity of foodscapes (Subgroup 1.1)	Public health, health geography, urban geography, sociology	USA/Canada (9), United Kingdom (6)	[13–26]
			Do foodscapes impact diet and health? Is healthy food less accessible for disadvantaged groups or neighborhoods?	Foodscape effects on diet (Subgroup 1.2)	Public health, health geography, urban geography	United Kingdom (9), USA/Canada (6)	[27–45]
			How does error risk affect food environment measurements?	Methodological suitability of foodscape databases (Subgroup 1.3)	Public health, health geography, geomatics	United Kingdom (2), Denmark (2)	[46–52]
Social and cultural approaches	Representations and material form of places and spaces linked to food, a socially constructed landscape	Interviews, focus group, observations, photos, drawings, maps	How do social and cultural factors (e.g. gender, race, socio-economic status migrations) shape food provisioning practices? How do people access food, perceive it and experience it?	Food access and structural inequalities (Subgroup 2.1)	Radical and social geography	USA/Canada (4), Thailand (2)	[11,53–64]
			How do culture-based food habits shape foodscapes?	Cultural and ethnic foodscapes (Subgroup 2.2)	Sociology, cultural geography, anthropology	USA/Canada (5), Sweden (3), Pacific islands (2)	[65–79]
			How are everyday food practices social constructions?	Everyday food practices as routines (Subgroup 2.3)	Ethnology, sociology, behavioral sciences	Scandinavian countries (4)	[80–89]
Behavioral approaches	The foodscape as physical, organizational, and sociocultural spaces in which clients/guests encounter food	Observations, interviews, focus group, reverse life- cycle analysis, document analysis, (advertisements, cook books), cross-sectional surveys, photos	What are the determinants of food behaviors in institutional out-of-home foodscapes?	Institutional foodscapes (Subgroup 3.1)	Education, behavioral sciences	Scandinavian countries (4), United Kingdom (2)	[90–98]
			How is food behavior affected by characteristics of domestic foodscapes?	Domestic foodscapes (Subgroup 3.2)	Architecture, sociology, marketing	Canada (2), Ireland (2)	[99–104]
			How do children become food consumers?	Retail foodscapes (Subgroup 3.3)	Sociology, marketing	Canada (2), Sweden (1)	[105–107]
				No specific	Food sciences	Europe (1)	[108]
Systemic approaches	The foodscape as a systemic concept close to the food system but pertaining to places linked to food	Interviews, phone surveys, internet searches, focus groups, ethnographic observations, document analysis (press releases and policies), photos, videos, autodriving, member- checking	How do alternative food networks shape foodscapes?	Local and ethical food networks (Subgroup 4.1)	Economic and political geography, rural sociology, environmental sciences	USA/Canada (5), United Kingdom (4)	[109–129]
			How do urban food policies shape foodscapes?	Urban food policies (Subgroup 4.2)	Economic and political geography	United Kingdom (7)	[130–140]
			How do foodscapes contribute to the identity of an event or a place?	Territorial marketing (Subgroup 4.3)	Tourism management, anthropology	Southern Europe (3), USA (2)	[10,141–149]
				No specific	Sociology	Global (1)	[150]

Hereunder, we present the four approaches according to the definitions of ‘foodscape’, the methods, and the research communities involved. For each approach, we distinguish subgroups of publications according to their objectives.

### Spatial approaches

Spatial approaches using statistics and GIS (geographical information systems) were the methodologies most frequently used to characterize the diversity of urban foodscapes and their impacts on diet and health, with a total of 40 publications.

In such studies, the term ‘foodscape’ designates a set of food outlets in an area (e.g. retail food shops, markets, restaurants, take-away restaurants, etc.) [17,32,33,47,48]. Scholars hence use the term ‘foodscape’ but principally refer to two definitions of food environment:

- The main study cited is Glanz *et al*. [6]. Authors using this spatial approach to foodscapes rely solely on Glanz’s first environment, the ‘community nutrition environment’ or what Lake et al. [47] call the “reality of the foodscape”.
- Another key citation is Winson [107], for whom ‘food environment’ encompasses “the multiplicity of sites where food is displayed for purchase and where it may also be consumed”.

Authors in public health, health geography, urban geography and sociology most often utilize this spatial approach to identify environmental determinants related to diet and health. More specifically, they study the spatial distribution (availability and accessibility) of food outlets. In general, food outlets are characterized as ‘healthy’ or ‘unhealthy’ according to the availability of fruits and vegetables.

This spatial approach category includes forty peer-reviewed articles published in twenty-nine journals, two of them publishing more than two of the articles: *Health & Place* (6) and *International Journal of Behavioral Nutrition and Physical Activity* (3). Of the forty publications, thirty-four are case studies, three are systematic reviews [35,44,48] and three are conceptual and position papers [33,36,39]. We identify three subgroups of publications, respectively characterizing foodscapes (typologies), examining their influence on diet, and assessing database quality (Table 1).

### Characterizing the diversity of foodscapes (Subgroup 1.1, Table 1)

The first subgroup of 14 publications focuses on the spatial distribution of different types of food outlets, in relation to inhabitants’ socioeconomic status. Their spatial unit of observation is a city or a neighborhood (medium-scale).

For instance, after a landscape characteristic assessment, visual observations and field surveys, Roe *et al*. [20] identified seven types of foodscapes in a British medium-sized city: “the landscape of the fast-food takeaway”, “the landscape of ethnic and ‘exotic’ food restaurants and shops”, “the industrialised supermarket landscape”.

Other papers are based on GIS methods. Most highlight the diversity of foodscapes people are exposed to, both in their neighborhood [18,22] and in their daily journeys [23]. By using participatory GIS to map foodscapes in three cities in Kenya, Ahmed *et al*. [26] shed light on food vendors and the food safety issues they face.

Four papers analyze the dynamics of foodscapes: Cummins *et al*. [14], Kolak *et al*. [24] and Filomena *et al*. [16] trace food retail structure changes over time respectively in Glasgow, Chicago and New York, while Chen and Clark [13] explore how food accessibility changes during a day in Franklin County, Ohio, USA.

### Foodscape effects on diet (Subgroup 1.2, Table 1)

In the second subgroup of 19 publications, scholars look at how foodscapes influence diet. Some also address the methodological question of how to define the best indicators to describe foodscapes when health issues are involved [38,43]. They develop place-based or person-based metrics relying on cross-sectional surveys and/or GIS methods. Person-based metrics are considered more appropriate by Chen and Clark [13]: they are used to define ‘foodscape exposure’, i.e. the types of foodscapes people are exposed to through their activity space. These studies show a positive association between exposure to high relative density of healthy outlets and high fruit and vegetable consumption, for instance in an English university [29] and in the five largest metropolitan areas of Canada [34]. Some, like Lebel *et al*. in Montreal [28], also find a positive association between exposure to fast-food restaurants through daily mobility and the risk of being overweight. However, foodscape influences on diet are not found to be significant in many studies. In particular, they seem to be sensitive to gender [34], to income [37], and also to the methods used to define foodscape exposure [28,34,43–45].

### Methodological suitability of foodscape databases (Subgroup 1.3, Table 1)

A third subgroup of 7 publications assess the suitability of the food-outlet databases employed to map food availability and accessibility. Burgoine [52] underlines “the time consuming negotiations, possible expense and probable stress of acquiring foodscape data from a robust source”. The validity of secondary sources of food-outlet data to describe foodscapes was tested in the United Kingdom [46,47,51], and in Denmark [49,50]. Lebel *et al*. [48] perform a systematic review and meta-analysis of such studies, showing greater variability in validity estimates in studies conducted in the USA than in Canada, the United Kingdom and Denmark. In particular, they find that studies focus on “global error in preliminary food environments data [but not on] how error affects the food environment measurements” [48]. They conclude that food outlet databases may be a valid way of characterizing foodscapes, despite minor errors in name or precise location, for instance.

To sum up, this first approach based on spatial analysis uses the term ‘foodscapes’ in the sense of Glanz’s community nutrition environment, to characterize and assess the physical food environment and its influence on diet. Both terms, ‘foodscape’ and ‘food environment’, are used in most papers. They appear interchangeable.

### Social and cultural approaches

The 38 publications in the second group use qualitative case studies and quantitative surveys of food procurement practices to show that foodscapes are not only part of a spatial environment but are also socially shaped. The authors explore the people-food-territory nexus in relation to cultural and social justice issues.

In this approach, ‘foodscape’ is clearly the preferred term. It is not interchangeable with ‘food environment’. The definition of ‘foodscape’ includes food outlets and all other places where people are exposed to food messages, such as house, school, or street [70]. It also includes the non-material environment, such as the media [85] and traditional food knowledge [72]. Hence, the ‘foodscape’ is not viewed only as a spatial distribution of food outlets (as in the spatial approach), but is considered the result of a place-based history including actors and policies. The term ‘foodscape’ is used “to emphasize the spatialization of foodways and the interconnections between people, food, and places” [60]. It is a “socially-constructed view of the field of food” [85]. “The foodscape concept both requires and rewards being situated in a particular place and focused on the relationships that a particular community has with food” [57].

This socio-cultural approach is critical of the spatial approach: the authors point out that it is the community and local history that determine whether a neighborhood becomes a food oasis or a food desert. They are generally in favor of studies linking foodscapes, socio-economic situation and health as a way of explaining health inequalities, and consider that the spatial approach is often too deterministic [57]. In particular, they underline the key role of culture in the food-people-place nexus [69], as well as the role of social relations [54]. For example, Panelli and Tipa [58] suggest the need to go beyond statistics and spatial analysis, which they consider decontextualize food. Their approach to the traditional behaviors of the Maori, whose health status is on average very poor, aims to “enable a better appreciation of the way well-being intersects with wider cultural practices”. These studies employ qualitative methods such as individual interviews, focus group interviews, and observations. They use photos, drawings and maps by interviewees themselves.

Of the 38 publications classified in this second approach, seven are books or book chapters and 31 are peer-reviewed articles published in 23 different journals, one of which published 5 of the papers: *Food*, *Culture & Society*. We identify three subgroups focusing respectively on structural inequalities, cultural and ethnic influences, and everyday food practices.

### Food access and structural inequalities (Subgroup 2.1, Table 1)

The first subgroup of 13 publications in critical geography and anthropology considers that food access issues result from structural inequalities affecting disadvantaged groups of people. The foodscape is used as a tool to highlight the roots of food injustice and insecurity. For example, Blake [53] shows how the processes that shape urban space also contribute to the production of food injustice in Hong Kong and in Singapore. Yasmeen [11,60] highlights how the modernization of the food system in Bangkok (rise of supermarkets) reverses the socio-spatial positions of women and men in favor of men. In the same vein, Hovorka [55] explores gender and food dynamics in Africa, proposing a feminist foodscapes framework. In such approaches, the foodscape is also used as a powerful lens through which to understand food procurement strategies [57]. Hammelman [54] observes that spatial proximity is not the major factor in food store choice by migrant women in Washington DC. This group of publications in critical geography and anthropology highlights the importance of multiple factors such as (i) accessibility without a car, (ii) safe traveling/walking, (iii) price of food, (iv) ‘daily activity spaces’, (v) the role of social status, and (vi) position in social networks and communities.

### Cultural and ethnic foodscapes (Subgroup 2.2, Table 1)

The second subgroup of 15 publications analyzes how cultural issues and migrations shape foodscapes. Coakley [68,69] with Polish immigrants in Ireland, Jochnowitz [70] with Russian-Jewish Americans in New York, Plank [73] with Thai Buddhist people in Sweden, and Cinotto [151] with Italian Americans in East New-York, highlight the ethnic adaptation of the foodscape according to the cultural needs of new populations. These ‘ethnic foodscapes’ help to shape the social identities of communities living in these neighborhoods. Contrastingly, Henderson and Slater [76] highlight the food practice acculturation of immigrants in Canada exposed to a new foodscape. In addition, Kwik [72] shows the influence of traditional food knowledge in social and cultural identity, and how the transmission of traditional food knowledge shapes the evolution of foodscapes. Bildtgård [65,66] uses the concept of mental foodscape to explain people’s choices of where to eat. His work reveals the hedonic relationships Swedish people have with food, the pictures conjured up for them by food from different countries.

### Everyday food practices as routines (Subgroup 2.3, Table 1)

The third subgroup of 10 publications explores the routine dimension of everyday food practices. Showing that places where snacks are usually consumed vary over time, and how they fit into people's daily lives, Syrjäla *et al*. [86] propose a new term ‘snackscape’ to characterize where and how people consume snacks [86]. Other scholars find that food practices like snacking begin during childhood [80], and can be influenced by food celebrities [85]. To explain this process, some authors focus on both actual and perceived foodscapes during childhood. How children perceive their foodscapes reveals “where consumers are generated” [81], that is to say, the cultural values and nutritional properties they associate with food [82], and their connection with nature [87].

To sum up, this second approach shows how neighborhoods’ foodscapes are shaped by social and cultural factors, such as social representations and specific food practices. Scholars explore this people-food-territory nexus as a system, not limited to a causal relationship between environment and individuals, which they would see as an overly deterministic approach. Unlike the first, spatial approach, studies do not focus on the presence of food in an environment defined as external to the consumer, or on the impacts of such an environment on individual practices. This second, socio-cultural approach focuses on how people access food, perceive it, and experience it, and how these practices and representations possibly influence/shape the foodscape. Scholars use the term ‘foodscape’ in preference to ‘food environment’. They include in this foodscape concept more than the material environment: individuals are part of the foodscape, as are policies and representations.

### Behavioral approaches

The third approach looks at how consumer perceptions of foodscapes explain and determine food behaviors and food learning.

Most of these behavioral studies rely on Mikkelsen’s definition [91]: “Foodscapes can be defined as physical, organizational and sociocultural spaces in which clients/guests encounter meals, food and food-related issues including health messages”.

We identify this behavioral approach in nineteen publications in fifteen journals, one of which published three of the papers: *Perspectives in public health*. The authors of the studies using a behavioral approach are researchers in the sociology of food, nutrition, anthropology, architecture, management, education, and marketing. With the exception of Verbeke *et al*. [108], who focus on the determinants of pork consumption, the publications can be classified by their focus on food behaviors in three different types of indoor foodscapes: institutional, domestic, and retail.

### Institutional foodscapes (Subgroup 3.1, Table 1)

The first subgroup of 9 publications focuses on understanding determinants of food behavior in institutional out-of-home foodscapes such as schools (including school gardens), workplaces, hospitals or prisons. These places form “environments to learn about food and nutrition” [90]. These authors use interviews, focus groups and observations as well as methods inspired by reverse life-cycle analysis [95]. Osowski *et al*. [92] show that in school, children learn social rules linked to food, such as commensality and nutritional knowledge. Such studies are particularly common in Scandinavian countries. For example, Torslev *et al*. [94] highlight how the context in which children eat influences their perception of the meal and also their eating practices. No understanding of foodscapes is possible without reference to the representations of those who experience and perceive them, such as children for school foodscapes [96].

### Domestic foodscapes (Subgroup 3.2, Table 1)

In the second subgroup of 6 publications, the term foodscape designates both social and physical home food characteristics. Studies are based on document analysis (cook books and advertisements), focus group interviews and quantitative surveys. Researchers study how these domestic foodscapes “shape daily food and cooking practices” [99]. For example, Kenneally [100,101] shows how the Irish ‘domestic foodscape’ changed in the mid-twentieth century, in particular with the introduction of the refrigerator, and how this affects foodways. Like Brembeck *et al*. [81], Le Bel and Kenneally [102] are interested in how food practices may be constructed during childhood. They find that young people’s memories of their domestic foodscape are centered on the kitchen, particularly the kitchen table. In two publications [103,104], the definition of ‘domestic foodscape’ is limited to the “view and/or appearance of an edible item that will be consumed” [103]. The authors focus on how food behavior is affected by different visual and objective characteristics of food and meals such as “size, shape, texture, colors”.

### Retail foodscapes (Subgroup 3.3, Table 1)

In the three publications with a retail focus, the term foodscape designates a store’s physical environment. These studies use focus group interviews and observations. Following the definition of Bitner [152], Lindberg *et al*. [106] consider that foodscape is “a complex mix of three environmental dimensions that influence consumers’ and employees’ responses and behaviors: ambient conditions […], space/function […], and signs, symbols and artifacts”. These authors view the foodscape concept as a way of exploring a set of characteristics (interior layout) of stores that may influence food choices. Lindberg *et al*. [106] analyze how choice of store layout influences stores’ sales and energy efficiency. Berry and McMullen [105] examine the impact of marketing on public health. Winson [107] analyzes what he calls the ‘spatial colonization’ of the supermarkets by ‘pseudo food’ (candies, potato chips, chocolate bars, soft drinks, etc.). He points out that “different spheres of the retail foodscape offer dramatically different nutritional options (and health implications) for consumers”.

In summary, this third approach highlights how food behaviors are influenced by individual perceptions of foodscapes in places. The unique feature of this behavioral approach lies in the scale of observation (specific indoor environments limited in size) and in the focus on individuals. Scholars show how environmental and individual factors interact. They may use both terms ‘foodscape’ and ‘food environment’ together, but Mikkelsen [91], identified as the major reference in this third approach, calls for preferential use of the term ‘foodscape’ when addressing “the relationship between food, its spatial context and the viewer”.

### Systemic approaches

The fourth and last approach (43 publications) is critical of the global corporate food regime, considering it unsustainable, and promotes alternative strategies of “reterritorialization or respatialization of food” [153]. These studies use the term foodscape as a synonym of ‘food system’ but pertaining to the places linked to food. For example, Fraser [112] and Lowitt [117] define the foodscape as the set of places and spaces linked to food throughout the food chain, from production to consumption. Roep and Wiskerke [123] use foodscape “to describe the spatial distribution of food across (urban) spaces and institutional settings”. This approach is well represented by the book *Worlds of Food*: *Place*, *Power and Provenance in the Food Chain* [154], which condemns the “placeless foodscapes” of the global industrialized food system and considers “local food systems” or “place-based foodscapes” as more sustainable alternatives [134]. Showing how the media shape food perceptions, Kautt uses the phrase ‘mediatized global foodscapes’ [150].

This approach includes 43 publications: 10 are books or book chapters and 33 are peer-reviewed articles published in 28 different journals. We distinguish three different subgroups covering local food networks, urban food policies and territorial marketing. With the exception of the Kautt cited above [150], which does not address these themes, the other 42 publications can be classified into these subgroups.

### Local and ethical food networks (Subgroup 4.1, Table 1)

The first subgroup, with 21 publications, focuses on local or alternative food networks (AFN) and on ethical dimensions of the foodscape (3 papers are from a special issue on ‘ethical foodscapes’ in *Environment and Planning A*, 2010). The main disciplines are economic and social geography, as well as rural sociology. Most papers (14 out of 21) are case studies based on qualitative methods: interviews, phone surveys, internet searches, focus groups, ethnographic observations and analysis of secondary data such as policy documents and press releases. Five papers are conceptual and position papers. Fraser's book [112] discusses food regimes shaping food production and consumption, while Mann’s book [128] argues that voting and participation are ways of contesting these power structures.

The authors highlight the potential of alternative/local (the two terms are often associated) food networks to meet the objectives of sustainable development. Research questions are related to how such networks can shape more sustainable food practices [118,123]. Sage [125] and Sharp [126] show how AFNs reconnect producers and consumers, rural and urban areas. Analyzing the political debate concerning the ‘sugar tax’ in Mexico, Fraser introduces the concept of ‘foodscapes of hope’ to “summarize new spatial formations regarding the production and consumption of food” such as AFNs [129]. Lowitt [117] claims that the foodscape lens helps to analyze changes in the social-ecological interactions that make up food systems and to identify mechanisms for promoting community food security. Carolan [109] shows that exposure to ‘alternative foodscape experiences’ may lead stakeholders “to create alternatives to the global food system”. This is confirmed by Psarikidou and Szerszynski [121] in England and by Rossi [124] in Italy, who note that AFNs could create ‘ethical foodscapes’. Goodman *et al*. [115] characterize such ‘ethical foodscapes’ as “a way of conceptualizing and engaging critically with the processes, politics, spaces, and places of the praxis of ethical relationalities embedded and produced in and through the provisioning of food”. Herman [116] and Richardson *et al*. [155] explain how ethical discourses about food are constructed. Freidberg [113] and Morgan [120] appeal for studies dealing with ‘ethical foodscapes’ to consider people who are not directly engaged in AFNs. Morgan [120] illustrates the diversity of ‘ethical foodscapes’ with three examples: carbon-labeling, school-food reform and the politics of care. Exploring the city region foodscape of rapidly growing Dar es Salaam, Wegerif and Wiskerke [127] call for more academic and policy attention to be paid to the middle-ground, neither global nor local, food systems that are delivering at city feeding scale.

### Urban food policies (Subgroup 4.2, Table 1)

The second subgroup of 11 publications focuses on public policies, in particular, urban policies supporting food relocalization. Disciplines and methods are the same as in the preceding subgroup: qualitative studies and position papers in sociology and geography.

These publications address the place of the “food question in theory, policy and practice” [119]. They show how public policies shape and frame foodscapes and how they carry visions of foodscapes [133]. Conversely, Potter and Westall [132] show how foodscapes can support policy orientations: they argue that food advertising and the media (cooking shows in particular) helped to implement the neoliberal policy of austerity in Great Britain.

The authors analyze—and call for—local food policies aimed at improving foodscapes’ sustainability, especially through food relocalization [134]. Cities are considered as the best scale to respond to the current food insecurity crisis, called by Morgan and Sonnino [131] a ‘new food equation’. Against a background of increasing community food initiatives, the city is depicted “as a site of social and ecological innovation/transition with respect to the food system” [130]. However, several limitations to urban food policies are identified, mainly concerning food production, food access and relationships between producers and consumers [131,133]. Urban food policies rarely encompass all the components, the stakeholders and the spaces in foodscapes. Local authorities also find it difficult to build inclusive and consensual food strategies [130,131,138].

### Territorial marketing (Subgroup 4.3, Table 1)

In this subgroup of 10 publications, foodscape refers both to agricultural landscapes (and their traditional food products), and to the place of food and beverages in festive environments/events. In this approach, food adds value to and shapes the identity of a place or an event. Researchers in tourism management, marketing, social anthropology and architecture use qualitative methods: observations and interviews, as well as “photography and videography, autodriving, and member checking” [142].

In seven publications, food is considered as a heritage which “contributes to the identity construction of a locality” as Pezzi underlines it [145]. This author, as well as Forné [143], Bjork [149] and Ron and Timothy [147], view some foodscapes as an opportunity to develop food tourism. They describe the cultural and environmental factors encouraging stakeholders to build territorial marketing strategies around culinary heritage. For example, Gavrilidou *et al*. [144] claim that foodscapes created by urban gardens play a role “in the reactivation of institutions and communities, a lesson of resilience in a city going through a deep crisis”.

In two publications, the authors use the term ‘foodscape’ to characterize festive environments involving food and beverages, like food festivals or the festivities that surround athletic events. They speak of the ‘ludic foodscape’ [142] or ‘festive foodscape’ [10]. To Adema [10], food is one of the characteristics which “matter for theming a locality, asserting differentiation through aggrandizement, securing a place brand, and concurrently generating senses of place and community”.

In summary, the term ‘foodscape’ is used in this fourth approach as synonymous with food system, to contest the power of the corporate food regime and to promote local, ethical, and sustainable food networks. In this approach, the definition of the foodscape is completely different from that of the food environment.

## Discussion

We systematically reviewed the scientific literature on the term ‘foodscape’.

Our results showed that the term has increasingly been used over the past decade in the English-speaking literature, in various disciplines, mainly in North America and Northern Europe. We distinguished four approaches: i) Spatial approaches use statistics and spatial analysis to characterize the diversity of urban foodscapes and their diet and health impacts; ii) Social and cultural approaches combine qualitative case studies and quantitative surveys of food procurement practices, showing that foodscapes are socially shaped, and highlighting structural inequalities in the food system; iii) Behavioral approaches focus on consumer perceptions of foodscapes as determinants of food behaviors and food education; iv) Systemic approaches consider the whole food system, denouncing the unsustainability of the global corporate food regime, and promoting local, ethical, and sustainable food networks.

In the following discussion, we show that these four approaches address different issues related to food security, at various scales. Then, according to the diverse definitions used (explicitly or implicitly) in our corpus, we discuss the polysemy of the term ‘foodscape’, its unique features and added-value compared to the term ‘food environment’. Finally, we indicate some perspectives for a research agenda.

### Foodscape issues and scales

Foodscape studies address three main food issues: public health, social justice, and the sustainability of food systems. They address such issues at various scales, from micro-scale to global scale. As shown in Table 2, each subgroup of publications identified in the results section includes publications at only one or two spatial scales. Publications within each subgroup focus on the same one or two food issues.

**Table 2:** Issues and scales covered by foodscape studies.

Issues Scales	Public health	Social justice	Sustainability of food systems
**Global**			Subgroup 4.1: Local and ethical food networks Subgroup 4.2: Urban food policies
**Neighborhood, city, region**	Subgroup 1.1: Characterizing the diversity of foodscapes Subgroup 1.2: Foodscape effects on diet Subgroup 1.3: Methodological suitability of foodscape databases		
		Subgroup 2.1: Food access and structural inequalities Subgroup 2.2: Cultural and ethnic foodscapes Subgroup 2.3: Everyday food practices as routines	
**Indoor environments**	Subgroup 3.1: Institutional foodscapes Subgroup 3.2: Domestic foodscapes Subgroup 3.3: Retail foodscapes		Subgroup 4.3: Territorial marketing

The first issue addressed by foodscape publications relates to public health, in line with international recommendations to take into account the impact of the food environment on food security [1]. Spatial and behavioral approaches explore the impact of the food environment on diet and health. Within these publications, the foodscape is examined at multiple scales: neighborhood and wider for the spatial approach, micro-scale of buildings and kitchens for the behavioral approach.

The second issue relates to social justice: spatial and socio-cultural approaches explore the definition of food security: “physical and economic access to sufficient, safe and nutritious food to meet their dietary needs and food preferences for an active and healthy life” [156]. Spatial approaches document social inequalities in physical access to healthy food while socio-cultural approaches reveal the multiple dimensions of food provisioning strategies (physical access, price, culturally appropriate food, opening hours, etc.), showing “the shifting boundaries of food environment” [54]. Some studies focus on specific segments of populations, by gender, race, socio-economic or migration status. Within the social justice subject area, the foodscape is examined at mezzo scales: neighborhoods, urban spaces used for individual mobility, and social relationship networks.

The third issue relates to the lack of sustainability of the global food system: systemic approaches denounce the impacts of globalization, challenge the capitalist food regime, and propose alternatives based on food relocalization, alternative food networks, tourism, heritage. The authors of these publications refer to foodscapes at multiple scales (local and global, physical and organizational) and often within a long-term perspective, linking the foodscape with the long-term trend in food regimes.

This variety of issues, considered at different scales, illustrates the richness of the concept, but also the polysemy of its definition, which is another finding from our review.

### Foodscape definitions

Definitions of foodscapes vary from material to more holistic and socio-cultural. All the authors include in ‘foodscape’ at least the physical spaces and places for selling (and eating) food—the actual sites where people can find food. Public health, nutritionist and geomatics scholars often consider only this “reality of the foodscape” [47]. Social science scholars add economic, socio-cultural, and political aspects to the definition of the foodscape. Those taking a socio-cultural or behavioral approach to the foodscape also incorporate the “organizational and sociocultural spaces” [91] that deliver food messages, such as the media, advertising, or public policies. Hence, our results show that the landscapes underpinning the studies vary. Not all scholars conceive the foodscape as “a marriage between food and landscape, both the conceptual notion (idea) of landscape and actual, physical landscapes” [10].

The results of our scoping review do not yield a strict distinction between the two terms ‘foodscape’ and ‘food environment’.

In the first approach, aimed mainly at building maps or indexes of food accessibility, or establishing statistical correlations between the spatial presence of food and individual behaviors, the two terms ‘foodscape’ and ‘food environment’ are synonymous. They are used interchangeably and with reference to Glanz’s community nutrition environment [6], mainly related to density, diversity, and proximity of food outlets. These studies share an environment-based, deterministic way of thinking, and seek to measure the impacts of environmental characteristics on individuals.

In contrast, in the three other approaches (social, behavioral and systemic), the two terms ‘foodscape’ and ‘food environment’ are not synonymous. Scholars consider the foodscape not as an environment external to individuals, but as a landscape including, perceived, and socially shaped by individuals. These approaches share a systemic way of thinking, considering culture and experience of food as part of the foodscape. Foodscape is the preferred term for addressing the complexity of the food system, from micro-scale individual practices to the global food regime.

Our results highlight the added-value of the foodscape concept in how food is examined in relation to the landscape. As underlined by Wegerif and Wiskerke [127], both material *and* social dimensions of foodscapes, and their interconnectedness, are in fact basic features of the “scapes” described by Appadurai [157]. This author adds to words like media, finance ideology, or technology, the “common suffix scape to indicate first of all that these are not objectively given relations which look the same from every angle of vision, but rather that they are deeply perspectival constructs, inflected very much by the historical, linguistic and political situatedness of different sorts of actors”. While he himself does not use the term ‘foodscape’, his insights on the role of a global cultural economy and on the tension between cultural homogenization and heterogenization are useful in analyzing how food is conceived, traded, grown, reared, processed, sold, and consumed in physical, organizational and socio-cultural spaces.

To sum up, the results of our scoping review suggest that researchers within the food environment field use the term ‘foodscape’ rather than ‘food environment’ to emphasize that the food around us is not just an objective reality (like the spatial distribution of food outlets), but is also a subjective “deeply perspectival construct”, to use Appadurai’s words. ‘Foodscape’ is the right term when explaining how food landscapes are shaped, influenced, transformed by social practices (shopping, cooking, eating), by political and legal institutions, by economic decisions, and by relations of power within food systems. ‘Foodscape’ should also be the preferred term when examining how food landscapes are perceived differently by each of us according to our “historical, linguistic and political situatedness”. The HLPE clearly includes such approaches in food environment studies [1]. Lytle and Sokol [158] consider that “it is also important to see the food environment as a construct in a larger, ecologically-based conceptual model”. We believe, however, that the growing interdisciplinary food environment field could use the term ‘foodscape’ as a distinctive concept focusing on the constructivist, political and perspectival dimensions of the ‘food environment’.

### Limitations

This review was limited to the term ‘foodscape’, seeking to clarify its use, definitions, and utility, and therefore does not encompass all food environment or food system studies. Other publications may contribute to each of the four approaches that we have distinguished, without being included in our corpus if they do not use the term ‘foodscape’. Further research may look at the similarity and distinctness of other terms used to describe the relationships between consumers and their food environment. Closely related terms include for instance the ‘edible landscape’, another term designating the spaces producing food within the city, in landscape and architectural design [159,160]. Moreover, there are almost no foodscape studies in the Latin countries of Europe [161], where scholars have long used other terms to tackle the relationship between space and food. Examples include ‘*terroirs’*, designating place-based authenticity of food production and processing [162], or ‘gastronomy’, highlighting the city- and region-specific cultural features of cuisines [163]. Future reviews could encompass these diverse concepts and the connections between them, to draw a wider picture of the relationships between consumers and their food environment in urban and rural areas.

### Conclusion: A research agenda

Foodscape research originated in awareness of the negative public health impacts generated by the global corporate food regime. Studies that consider food environments as a determining factor of individual behaviors have however been criticized for focusing “more on creating environments that promote healthy choices than on the political and economic decisions which shaped these environments to begin with” [164]. In response, scholars have explained how food deserts emerge over time [165–167], and showed that creating a new and healthier urban food landscape operates as a spatialized form of ‘neoliberal paternalism’, assuming that “the poor people do not have the skills to manage their own affairs” [168]. From food deserts to foodscapes, our review shows that research has shifted from a physical approach towards a more holistic perspective involving social, spatial, and temporal approaches. For instance, Hammelman [54] uses the foodscape as a “lens for drawing attention to the relational nature of food provisioning strategies that can be constrained by the built environment by demonstrating the ways these strategies are both social and mobile”. Thus, summarizing the relationship between foodscape and diet, Clary *et al*. [33] argue that “the decision to opt for a specific outlet type relies on both the discrete moments when individuals willing to use the foodscape consider their latitude to acquire food (i.e. they assess accessible opportunities) and the way individuals interpret and react to foodscape exposures over the life course”. They therefore suggest a research agenda: (i) taking on board people-based activity spaces to better define foodscape exposure through their daily mobility; (ii) developing “qualitative investigations on perceptions of the [food] environment” for “questioning the normative influences of the foodscape on the intention to utilize healthy and unhealthy outlets”; and (iii) moving from cross-sectional designs to natural experiments and longitudinal designs.

This could be achieved by applying integrated and multiscale approaches, as well as action-oriented studies. Foodscape studies also require interdisciplinary frameworks involving researchers in spatial modeling, social sciences, public health, and urban planning. All this should contribute to the further development of foodscape analysis as a tool for urban planning and community development.

Another insight from our review is the benefit of combining material and physical, perspectival and political dimensions. Foodscapes have a material or physical dimension illustrated by the spatial distribution of food outlets, their inclusion in urban patterns, and by the diversity of food places: indoors or outdoors, small or large, specialized or diversified. Foodscapes also have a perspectival dimension: they are places and ways differently perceived and used by consumers according to modes of travel, social relations networks, food culture, gender issues, security concerns, etc. Finally, foodscapes have a political dimension: they partially depend on national and local policies, and food is increasingly taken into account in local policies, zoning codes, and strategic or comprehensive plans [169]. A challenge for future research is to understand the multiple connections between the food environment, consumers’ spatial practices, and local public actions. While Bivoltsis *et al*. [170] call for “future studies to consider the comparative effect on dietary outcomes of spatial exposure measures derived from place-based versus people-based approaches”, we advocate interdisciplinary research on foodscapes combining place-based and people-based approaches at different scales. Recent publications show how social practices theory could come in, helping to explore how foodways and foodscapes co-evolve in the long term [171–173]. Without using the term foodscape, Lytle and Sokol also call for more interdisciplinary work on food environments [158] and Pitt *et al*. [4] consider that “greater emphasis on how individual and environmental factors interact in the food environment field will be key to developing stronger understanding of how environments can support and promote healthier food choices”.

To conclude, this scoping review provides an updated overview of scientific publications using the term ‘foodscape’. Within the food environment field, ‘foodscape’ could be a distinctive concept for studies focusing on the constructivist, political, and perspectival dimensions of food security issues. ‘Foodscape’ should hence be the preferred term when tackling not only a physical food environment, but also a socially constructed landscape, perceived differently according to the stakeholder’s background and situation. Following the systemic approaches to food systems, people-based and place-based interdisciplinary research is needed to explore this food-people-territory nexus.

## Supporting information

S1 Checklist Preferred reporting Items for systematic reviews and meta-analyses extension for scoping reviews (PRISMA-ScR) checklist. (DOCX)

S1 Table (XLSX)
